# Erie County Medical Society

**Published:** 1866-01

**Authors:** 


Erie County Medical Society.—The regular Annual Meeting of the Erie County Medical Society was held in this city Tuesday, 9th inst., when the following officers were elected for the ensuing year:

President, George Abbott, M. D.; Vice President, J. R. Lothrop, M. D.; Secretary, Thomas M. Johnson, M. D.; Treasurer, Wm. Ring, M. D.; Librarian, J. B. Samo, M. D.; Primary Board—L. P. Dayton, M. D., E. B. Tifft, M. D., H. Vanguisling, M. D.; Censors—S. W. Wetmore, M. D., S. F. Mixer, M. D., J. R. Lothrop, M. D., P. H. Strong, M. D., J. Hauenstein, M. D.; Delegates to the State Medical Society—J. F. Miner, M. D., J. R. Lothrop, M. D., C. C. Wyckoff, M. D., Thos. F. Rochester, M. D.

